# Kindlin-2 controls TGF-β signalling and Sox9 expression to regulate chondrogenesis

**DOI:** 10.1038/ncomms8531

**Published:** 2015-07-07

**Authors:** Chuanyue Wu, Hongli Jiao, Yumei Lai, Wei Zheng, Ka Chen, Hong Qu, Weimin Deng, Pingping Song, Ke Zhu, Huiling Cao, Deborah L. Galson, Jie Fan, Hee-Jeong Im, Yujie Liu, Ju Chen, Di Chen, Guozhi Xiao

**Affiliations:** 1 Department of Pathology, University of Pittsburgh, Pittsburgh, Pennsylvania 15261, USA.; 2 Department of Biology and Shenzhen Key Laboratory of Cell Microenvironment, South University of Science and Technology of China, Shenzhen 518055, China.; 3 Department of Biochemistry, Rush University Medical Center, Chicago, Illinois 60612, USA.; 4 Department of Medicine, University of Pittsburgh, Pittsburgh, Pennsylvania 15240, USA.; 5 Department of Surgery, University of Pittsburgh, Pittsburgh, Pennsylvania 15240, USA.; 6 Department of Medicine, University of California San Diego, La Jolla, California 92093, USA.

## Abstract

The signals that control skeletogenesis are incompletely understood. Here we show that deleting Kindlin-2 in Prx1-expressing mesenchymal progenitors in mice causes neonatal lethality, chondrodysplasia and loss of the skull vault. Kindlin-2 ablation reduces chondrocyte density by decreasing cell proliferation and increasing apoptosis, and disrupts column formation, thus impairing the formation of the primary ossification center and causing severe limb shortening. Remarkably, Kindlin-2 localizes to not only focal adhesions, but also to the nuclei of chondrocytes. Loss of Kindlin-2 reduces, while the overexpression of Kindlin-2 increases, *Sox9* expression. Furthermore, the overexpression of Sox9 restores the defects in chondrogenic differentiation induced by Kindlin-2 deletion *in vitro*. In addition, Kindlin-2 ablation inhibits TGF-β1-induced Smad2 phosphorylation and chondrocyte differentiation. Finally, deleting Kindlin-2 in chondrocytes directly impairs chondrocyte functions, resulting in progressive dwarfism and kyphosis in mice. These studies uncover a previously unrecognized function for Kindlin-2 and a mechanism for regulation of the chondrocyte differentiation programme and chondrogenesis.

## Abstract (web summary)

The Kidlins are proteins found in cell focal adhesion sites where they regulate integrins, and in the nucleus where their role is unknown. Here the authors show that Kindlin-2 controls chondrogenesis by regulating integrin b1 activation and Sox9 and TGF-β nuclear signalling.

## Graphical abstract

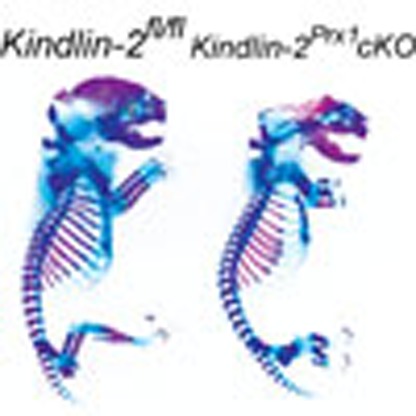

In vertebrates, the skeleton not only confers morphology, but also provides essential functions of life, including control of movements and protection of vital organs. During embryonic development, the skeleton is formed from mesenchymal stem cells (MSCs) through two distinct mechanisms—intramembranous and endochondral ossification. In intramembranous ossification, which forms flat bones, including the skull vault and part of the clavicle, MSCs condense and differentiate into osteoprogenitors, and later, osteoblasts^1^. In endochondral ossification, which forms most of the other bones, including all long bones and vertebrae, an intermediate cartilaginous template is first formed through a process that involves MSC condensation, chondrocyte proliferation, hypertrophy and apoptosis; this is followed by replacement by bone in the adjacent metaphysis through multiple steps, including new blood vessel invasion, osteoclast differentiation and digestion of the calcified cartilage and osteoblastogenesis from the perichondrial cells and bone formation^2^. Endochondral ossification contributes to the longitudinal growth of the skeleton. A better understanding of molecular mechanisms controlling skeletogenesis and homeostasis is critical for the development of new strategies to treat major skeletal diseases, such as osteoarthritis.

During skeletal development and homeostasis, chondrocyte differentiation is tightly controlled by key transcription factors, as well as distinct signalling pathways. Sox9, a transcription factor of the sex-determining region Y (SRY)-related high mobility group box family of proteins, is essential for converting MSCs into chondrocytes and regulates multiple functions of chondrocytes, such as proliferation, survival, differentiation and hypertrophy^3^,^4^,^5^,^6^. Sox9 controls chondrocyte differentiation by directly activating chondrogenic gene expression (for example, type II collagen or Col2a1)^7^,^8^ and through indirect upregulation of Sox5 and Sox6 (ref. 3), both key transcription factors that regulate chondrocyte differentiation^9^. Transforming growth factor-β (TGF-β) signalling is a fundamental route that controls multiple properties of chondrocytes and skeletal development and homeostasis^10^,^11^,^12^,^13^,^14^,^15^,^16^,^17^,^18^. While the importance of Sox9 and TGF-β signalling in the chondrocyte differentiation programme is well documented in the literature, the mechanism of their modulation during this process is poorly understood.

During skeletal development and homeostasis, bone cells or their precursors must migrate to appropriate locations and communicate with their extracellular matrix to form distinct elements of the skeleton. Kindlins are a family of evolutionarily conserved cytoplasmic proteins that are emerging as key regulators of integrin-mediated cell–extracellular matrix adhesion, migration and signalling^19^,^20^,^21^,^22^,^23^,^24^,^25^,^26^. In mammals, the Kindlin family consists of three members, Kindlin-1, -2 and -3, each of which contains a FERM (F for 4.1 protein, E for ezrin, R for radixin and M for moesin) domain that is responsible for interacting with β-integrin cytoplasmic tails. Mutations in the *KINDLIN-1* gene lead to the Kindler syndrome in humans, which is characterized by skin blistering^27^,^28^. Mutations in the *KINDLIN-3* gene impair integrin activation in humans and result in leukocyte adhesion deficiency-III, severe bleeding, frequent infections and osteopetrosis^29^,^30^,^31^,^32^. Global inactivation of *Kindlin-2* in mice resulted in early embryonic lethality at E7.5 (ref. 33). Because of this finding, and because there are no human genetic diseases known to be linked to mutations in the *KINDLIN-2* gene, functions of Kindlin-2 in regulating the development and homeostasis of specific organs and tissues are largely unknown.

To determine the roles of Kindlin-2 during skeletogenesis, we use a conditional knockout strategy to selectively ablate Kindlin-2 expression in two distinct skeletogenic cells at different stages of skeletal development in mice. We perform comprehensive analyses of two mouse models in which Kindlin-2 is ablated in limb and head mesenchymal progenitor cells or chondrocytes. We demonstrate that conditional ablation of Kindlin-2 in Prx1-expressing limb and head mesenchymal progenitors in mice results in neonatal lethality, severe chondrodysplasia and complete loss of the skull vault. Loss of Kindlin-2 inhibits chondrocyte proliferation, increases chondrocyte apoptosis and disrupts the column formation, which together impairs formation of the primary ossification centre (POC) of the long bones and results in limb and digit shortening. Thus, Kindlin-2 expression in mesenchymal progenitors is critical for endochondral ossification. Kindlin-2 expression is also required for control of intramembranous ossification, as demonstrated by a complete loss of the skull vault and severe hypoplastic clavicles in the mutant mice. Of particular significance, we find that Kindlin-2 localizes to not only focal adhesions, but also to the nuclei of chondrocytes where it activates Sox9 expression, a master regulator of chondrogenesis. Overexpression of Sox9 restores the defects in chondrogenic differentiation induced by Kindlin-2 deletion *in vitro*. We further demonstrate that Kindlin-2 ablation inhibits TGF-β1-induced Smad2 phosphorylation and chondrocyte differentiation. Finally, deleting Kindlin-2 in chondrocytes directly impairs chondrocyte functions, resulting in progressive dwarfism and kyphosis and osteopenic phenotype in mice. Collectively, our results shed important new light on the functions of Kindlin-2 and its mode of action in skeletogenesis.

## Results

### Multiple skeletal abnormalities in *Kindlin-2* ^*Prx1*^ cKO mice

**Figure 1:**
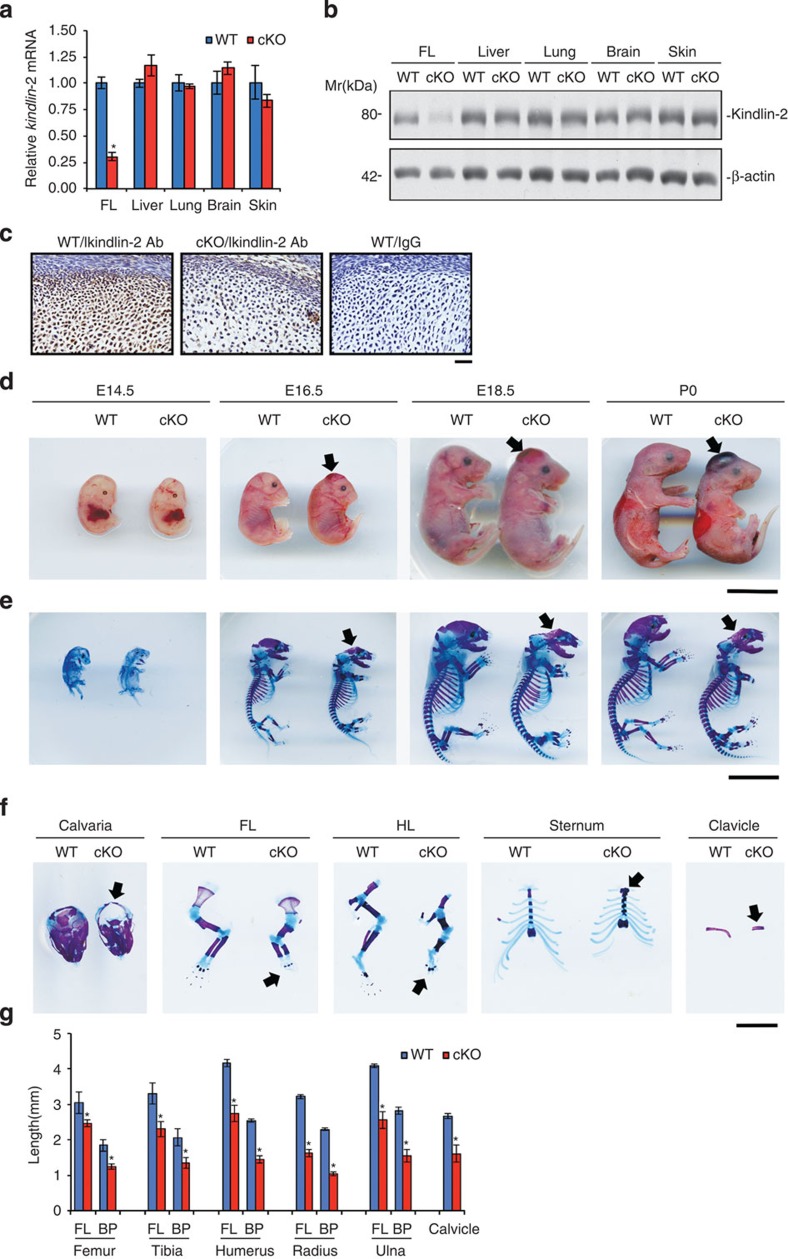
*Kindlin-2*^*Prx1*^ cKO mice display multiple striking skeletal abnormalities. (**a**,**b**) Quantitative qPCR and western blot analyses. Total RNA or protein extracts isolated from the indicated tissues of E14.5 *Kindlin-2*^*Prx1*^ cKO (cKO) or Cre-negative *Kindlin-2*^*fl/fl*^ (WT) littermates were subjected to qPCR (**a**) or western blot analyses (**b**) for Kindlin-2 expression. FL, forelimb. *Kindlin-2* mRNA was normalized to *Gapdh* mRNA. β-Actin was used as a loading control. Experiments were repeated three times. *P<0.05, versus WT, Student’s *t*-test. Results were expressed as mean±s.d. (**c**) Immunohistochemistry. Sections of E14.5 WT and *Kindlin-2*^*Prx1*^ cKO humeri were stained with anti-Kindlin-2 antibody or normal IgG. Note: Kindlin-2 protein is strongly detected in the nuclei of WT chondrocytes, but is dramatically reduced in mutant cells. Scale bar, 40 μm. (**d**) Gross appearance of E14.5–P0 *Kindlin-2*^*Prx1*^ cKO and WT embryos showing a haematoma on the top of the head of mutants (arrowhead). Scale bar, 0.5 cm. (**e**) Alizarin red and alcian blue double stain of E14.5–P0 skeletons. Scale bar, 0.5 cm. (**f**) Calvaria, FL, hindlimb (HL), sternum and clavicle from E18.5 embryos. Complete loss of the skull vault, shortened and broadened scapula, long bone and sternum, hypoplastic clavicle, and blocked (FL) or delayed (HL) elongation of distal phalanges are seen in mutants. Scale bar, 0.5 cm. (**g**) Quantitative data from F. *N*=4, *P<0.05, versus WT, Student’s *t*-test. Results were expressed as mean±s.d.

To investigate the potential roles of Kindlin-2 in the regulation of skeletogenesis, we used Cre-Lox technology to conditionally delete Kindlin-2 expression in limb and head mesenchyme. *Kindlin-2*^*fl/fl*^ mice, in which exons 5 and 6 of *Kindlin-2* gene are flanked by loxP sites, were generated as described in Methods (Supplementary Fig. 1). Crossing the *Prx1-Cre* transgenic mice^34^ with the *Kindlin-2*^*fl/fl*^ mice generated *Prx1-Cre; Kindlin-2*^*fl/+*^mice. Further cross-breeding of the *Kindlin-2*^*fl/fl*^ mice with *Prx1-Cre; Kindlin-2*^*fl/+*^mice successfully produced *Prx1-Cre; Kindlin-2*^*fl/fl*^ mice, the limb and head mesenchyme conditional KO mice (referred to as *Kindlin-2*^*Prx1*^cKO hereafter). *Prx1-Cre* mice, in which the 2.4-kb *Prx1* (paired-related homeobox gene-1) gene promoter drives Cre expression in limb and head mesenchyme, have been widely used^34^. In these mice, Cre expression first appears at E9.5 in the forelimb mesenchyme and E10.5 in the hindlimb bud. These mice also express high levels of Cre protein in the head mesenchyme^34^. Quantitative real-time RT-PCR (qPCR) and western blot analyses revealed that Kindlin-2 expression was dramatically reduced at both the mRNA and protein levels in *Kindlin-2*^*Prx1*^cKO limbs, compared with those of Cre-negative *Kindlin-2*^*fl/fl*^ limbs (referred to as WT hereafter; Fig. 1a,b). Notably, loss of Kindlin-2 did not significantly alter the levels of Kindlin-1 and Kindlin-3 proteins in limbs (Supplementary Fig. 2). Immunohistochemical staining of humeral sections of the two genotypes showed high Kindlin-2 expression in WT chondrocytes, which was strikingly reduced in mutant chondrocytes (Fig. 1c). It is interesting to note that although Kindlin-2 is known as a cytoplasmic protein located at focal adhesion sites, it was strongly detected in the nuclei of chondrocytes (Fig. 1c). Strikingly, all mutant mice (>50) died immediately after birth. As shown in Fig. 1d, all mutants (>100) displayed a haematoma on top of the head, which grew larger over time. Alcian blue and alizarin red double staining of whole-mount skeletons revealed that, although both bone and cartilage were present, all mutants exhibited multiple striking skeletal defects (Fig. 1d–g and Supplementary Fig. 3), including: (1) severe forelimb and hindlimb shortening; (2) impaired digit elongation; (3) shortened, broadened and fused sterna; (4) complete loss of the skull vault, which may have contributed to the neonatal lethality due to lack of protection of brain tissue from damage during birth; and (5) severe hypoplasia of the clavicle, a defect observed in Runx2 haploinsufficiency in mice and the human disorder cleidocranial dysplasia^35^,^36^. Defects (1)–(3) suggest that Kindlin-2 is essential for endochondral ossification and defects (4) and (5) demonstrate that Kindlin-2 is critically involved in the regulation of intramembranous ossification, which forms the skull vault and part of the clavicle. Quantitative analyses revealed that the length of all the long bones (femur, tibia, humerus, radius and ulna) and their respective bony portions  stained red by alizarin red were significantly reduced in mutants (*P*<0.05, Student’s *t*-test; Fig. 1g). It should be noted that, although there were striking skeletal phenotypes, the overall body size of the mutants was not markedly different from that of the WT (Fig. 1d). The Kindlin-2 flox heterozygotes that harbour *Prx1-Cre* (that is, *Prx1-Cre; Kindlin-2*^*fl/+*^) were viable and fertile and did not show any marked skeletal phenotypes (Supplementary Fig. 3 and Fig. 1b,c).

### Kindlin-2 controls chondrocyte function and POC formation

**Figure 2:**
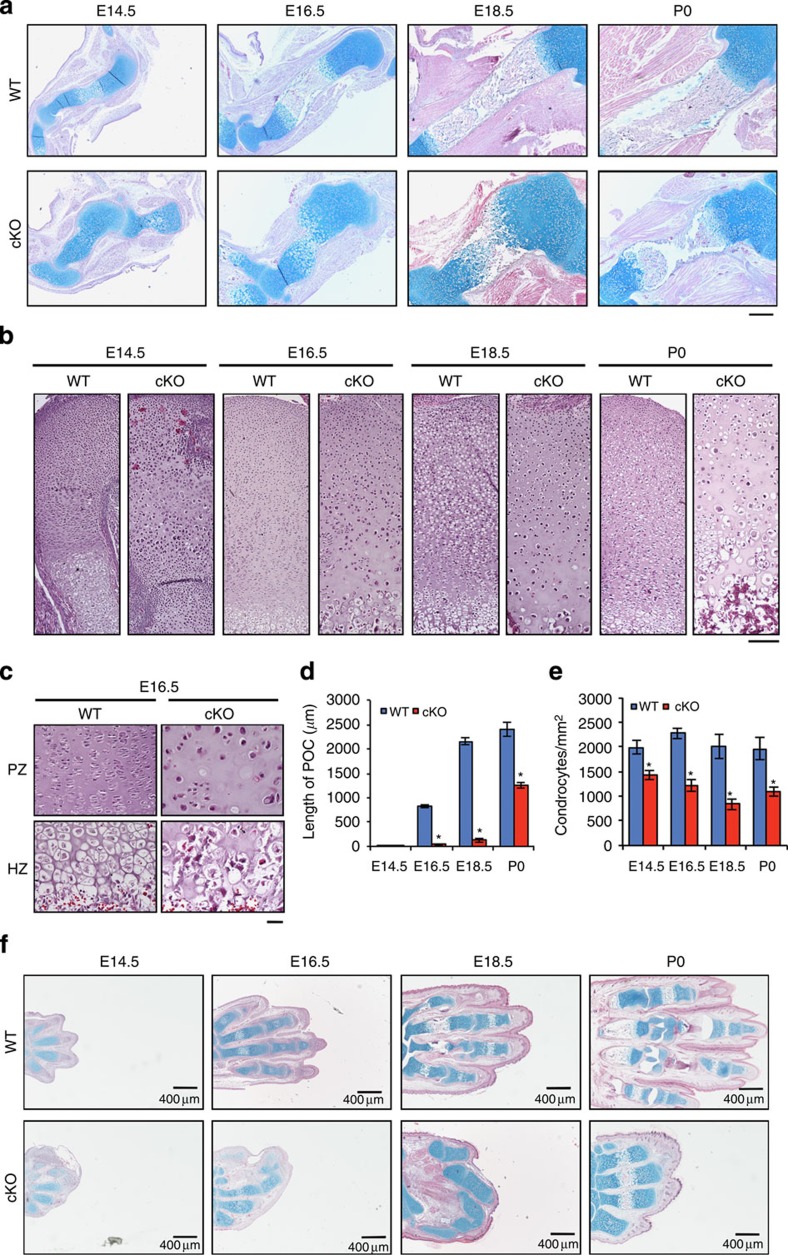
Reduced cellularity and disrupted column formation in *Kindlin-2*^*Prx1*^ cKO. (**a**) Alcian blue stain of humeral sections from Cre-negative *Kindlin-2*^*fl/fl*^ (WT) and *Kindlin-2*^*Prx1*^ cKO (cKO) mice shows severe delay in POC formation in mutants. Original magnification: × 40. Scale bar, 400 μm. (**b**) Haematoxylin staining of WT and *Kindlin-2*^*Prx1*^ cKO humeri. Original magnification: × 100. Scale bar, 160 μm. (**c**) Representative images of the proliferative zone (PZ) and the hypertrophic zone (HZ) of E16.5 WT and *Kindlin-2*^*Prx1*^ cKO humeri shows failure of column formation in mutants. Original magnification: × 400. Scale bar, 40 μm. (**d**) Quantitation of lengths of humeral POCs from **a**, *N*=4, *P<0.05, versus WT, Student’s *t*-test. Results were expressed as mean±s.d. (**e**) Quantitation of chondrocyte density of humeral growth plates in **b**. *N*=4, *P<0.05, versus WT, Student’s *t*-test. Results were expressed as mean±s.d. (**f**) Alcian blue stain of WT and *Kindlin-2*^*Prx1*^ cKO forelimb digits shows blocked elongation of digits and delayed formation of ossification centres in the phalange bones. Scale bar, 400 μm.

In mice, limb mesenchymal condensation occurs at around E11.5–E12.5 (ref. 37). After condensation, the majority of mesenchymal progenitor cells become chondrocytes that go into the proliferation and differentiation programme^37^. As shown in Fig. 2a, at E14.5, chondrocytes in the centre of the cartilage mould of the WT humerus enlarged and formed a highly organized hypertrophic zone, which was not observed in the mutant. By E16.5, a POC was formed in the middle of WT, but not mutant, humeri (Fig. 2a). At E18.5, the POC was partially formed in the mutant (Fig. 2a). At birth (P0), a significantly shorter POC was formed in the mutant (Fig. 2a,d). Loss of Kindlin-2 similarly delayed formation of ossification centres in digits (Fig. 2f). One major cellular defect in the mutant growth plate was a dramatic reduction of chondrocyte density starting at E14.5 and worsening over time (Fig. 2b,e and Supplementary Fig. 4). Furthermore, loss of Kindlin-2 greatly impacted chondrocyte size, morphology and organization in the growth plate. As shown in Fig. 2c and Supplementary Fig. 4, in the proliferative zone of the WT humeral growth plate, chondrocytes were discoid and arranged into regular columns, like stacks of coins, which were parallel to the long axis of the cartilage. In contrast, mutant chondrocytes were large and spherical, and failed to form columns (Fig. 2b,c). Instead of forming the highly organized hypertrophic zone in the middle of long bones, as found in the WT, the large spherical mutant chondrocytes were scattered throughout the growth plate (Fig. 2b).

### Kindlin-2 deletion reduces chondrocyte proliferation and survival

**Figure 3:**
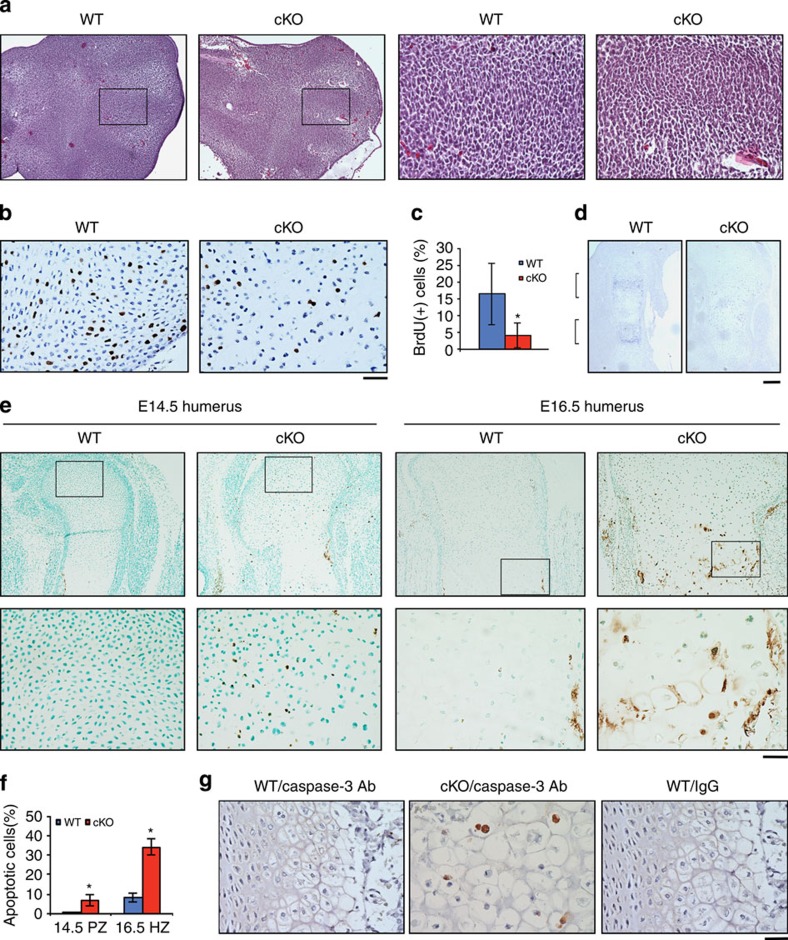
Kindlin-2 is critical for chondrocyte proliferation and survival. (**a**) Haematoxylin stain of E12.5 Cre-negative *Kindlin-2*^*fl/fl*^ (WT) and *Kindlin-2*^*Prx1*^ cKO (cKO) forelimb bud sections shows normal mesenchymal condensation and cell density in mutants. Original magnification: × 100 (two left lanes), × 400 (two right lanes). Scale bar, 160 μm (right), 40 μm (left). (**b**) BrdU staining of E14.5 WT and *Kindlin-2*^*Prx1*^ cKO humeral sections. Original magnification: × 400. Scale bar, 40 μm. (**c**) Quantitation of BrdU-positive cells from **b**. *N*=4, *P<0.05, versus WT, Student’s *t*-test. (**d**) *In situ* hybridization. E14.5 WT and *Kindlin-2*^*Prx1*^ cKO humeral sections were hybridized with an antisense riboprobe for *Ihh*. Scale bar, 160 μm. (**e**) TUNEL stain of E14.5 (left) and E16.5 (right) WT and *Kindlin-2*^*Prx1*^ cKO humeral sections. Representative lower magnification images ( × 100, top) and higher magnification images of the boxed areas ( × 400, bottom) are shown. Scale bar, 160 μm (top), 40 μm (bottom). (**f**) Quantitation of apoptotic cells of the proliferative zones (PZ) and hypertrophic zones (HZ). *N*=4, *P<0.05, versus WT, Student’s *t*-test. Results were expressed as mean±s.d. (**g**) Immunohistochemical staining of E14.5 WT and *Kindlin-2*^*Prx1*^ cKO humeral sections for cleaved (active) caspase-3 in chondrocytes. Scale bar, 40 μm.

As shown in Fig. 3a, at E12.5, mesenchymal condensation occurred normally and cell density was not reduced in the mutant limb buds. Results from *in vivo* BrdU (5-bromo-2-deoxyuridine) labelling experiments revealed that the chondrocyte proliferation rate was significantly reduced in the E14.5 mutant relative to WT growth plates (Fig. 3b,c). Indian hedgehog (Ihh) is known to play a critical role in the regulation of chondrocyte proliferation^38^,^39^,^40^,^41^. In situ hybridization of E14.5 humeral sections showed that *Ihh* mRNA expression was detected in pre-hypertrophic chondrocytes in WT growth plates, but was markedly reduced in mutant cells (Fig. 3d). Terminal deoxynucleotidyl transferase dUTP nick end labeling (TUNEL) staining of E14.5 humeral sections of the two genotypes demonstrated that chondrocyte apoptosis in the proliferative zone was dramatically increased in mutant growth plates (Fig. 3e,f). At E16.5, the apoptosis of hypertrophic chondrocytes was also greatly increased in the mutants (Fig. 3e,f). Note that basal apoptosis levels were increased in E16.5 compared to E14.5 chondrocytes in both genotypes. Consistent with increased cell apoptosis, cleaved (active) caspase-3 was upregulated in mutant chondrocytes compared with that of WT cells (Fig. 3g). Thus, reduced cell proliferation capacity and accelerated cell death could be two major reasons for the reduction of chondrocytes in mutant growth plates.

### Kindlin-2 regulates *Sox9* gene expression in chondrocytes

**Figure 4:**
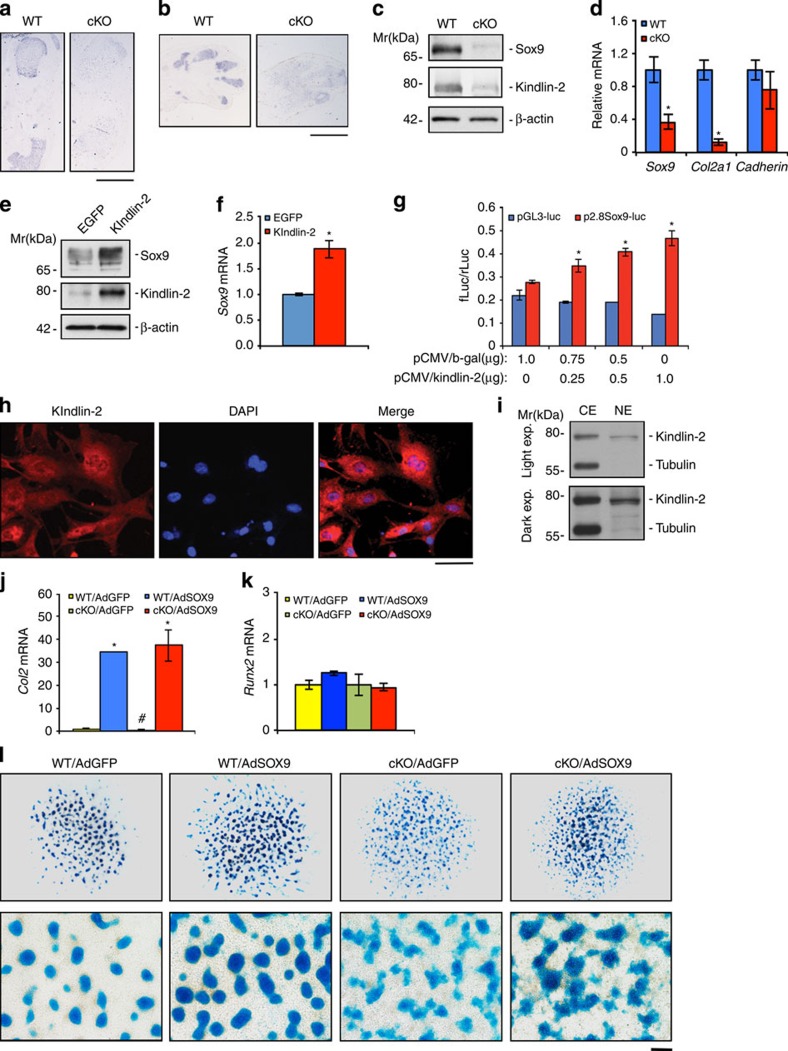
Kindlin-2 regulates Sox9 expression in chondrocytes. (**a**,**b**) *In situ* hybridization. E16.5 Cre-negative *Kindlin-2*^*fl/fl*^ (WT) and *Kindlin-2*^*Prx1*^ cKO (cKO) humeral (**a**) and forelimb digit (**b**) sections hybridized with antisense riboprobes for *Sox9*. Original magnification: × 40. Scale bar, 400 μm. (**c**) Protein extracts from E16.5 WT and *Kindlin-2*^*Prx1*^ cKO forelimbs used for western blotting for Sox9 expression. (**d**) Primary mesenchymal progenitors isolated from E13.5 *Kindlin-2*^*Prx1*^ cKO and WT limbs were differentiated for 4 days and analysed by qPCR for *Sox9*, *Col2a1* and *cadherin* expression, normalized to *Gapdh* mRNA. Experiments were repeated three times. *P<0.05, versus WT, Student’s *t*-test. Results were expressed as mean±s.d. (**e**,**f**) ATDC5 chondrocytes were infected with equal amounts of adenoviral vectors for enhanced green fluorescent protein (Ad/EGFP) or Ad/Kindlin-2, followed by western blotting (**e**) or qPCR analyses (**f**) for Sox9 expression. Experiments were repeated three times. *P<0.05, versus WT, Student’s *t*-test. Results were expressed as mean±s.d. (**g**) COS-7 cells were co-transfected with p2.8Sox9-luc, pRL-SV40 (for normalization), and the indicated amounts of pCMV/Kindlin-2 or pCMV/β-gal, followed by dual-luciferase assays. The amount of plasmid DNA was balanced as necessary with pCMV/β-gal such that the total DNA was constant for each group. Experiments were repeated three times. *P<0.05 versus 0 μg expression vectors, Student’s *t*-test. (**h**) Primary chondrocytes from E13.5 WT ribcages stained with an anti-Kindlin-2 antibody or 4′,6-diamidino-2-phenylindole, followed by fluorescence (IF) microscopy for localization of Kindlin-2. Scale bar, 50 μm. (**i**) Cytoplasmic (CE) and nuclear extracts (NE) from ATDC5 cells subjected to western blotting for Kindlin-2 and tubulin (a cytoplasmic protein). (**j**–**l**) Primary mesenchymal progenitor cells isolated from E13.5 *Kindlin-2*^*Prx1*^ cKO and WT limbs were infected with Ad/Sox9 or an equal amount of Ad/GFP. Four days later, the cells were cultured in the presence of 2 ng ml^−1^ TGF-β1 for another 6 days, followed by qPCR analyses for *Col2a1* (J) and *Runx2* (K), or alcian blue staining (L). mRNA levels were normalized to *Gapdh* mRNA. *P<0.05 (versus WT/AdGFP or cKO/AdGFP, Student’s *t*-test). #*P*<0.05 (versus WT/AdGFP, Student’s *t*-test). Experiments were repeated in triplicates. Scale bar, 800 μm (top), 160 μm (bottom). Results were expressed as mean±s.d.

*In situ* hybridization showed that the mRNA level of Sox9, a master regulator of chondrogenesis, was reduced in mutant humeri and digits compared with that of WT (Fig. 4a,b). Western blot analysis confirmed that Sox9 was reduced at the protein level in mutant limbs (Fig. 4c). qPCR analysis showed that the loss of Kindlin-2 reduced *Sox9* and its downstream target *Col2a1* mRNA expression without affecting the expression of mRNA for cadherin, a molecule required for mesenchymal chondrogenic condensation^10^ (Fig. 4d). In contrast, the adenoviral overexpression of Kindlin-2 in ATDC5 chondrogenic cells increased the levels of Sox9 protein (Fig. 4e) and mRNA (Fig. 4f) and activated 2.8-kb mouse *Sox9* gene promoter activity in a dose-dependent manner (Fig. 4g). Interestingly, although Kindlin-2 is widely recognized as a cytoplasmic protein, it was strongly detected in the nuclei of primary ribcage chondrocytes, as measured by immunofluorescence staining followed by confocal analysis (Fig. 4h). Western blot analysis using nuclear and cytoplasmic extracts showed that Kindlin-2 protein existed in the nuclei of ATDC5 cells (Fig. 4i). The adenoviral overexpression of Sox9 largely restored the defects in *Col2a1*, but not *Runx2*, mRNA expression (Fig. 4j,k) and chondrogenic nodule formation in micromass cultures (Fig. 4l).

### Loss of Kindlin-2 impairs TGF-β1 signalling

**Figure 5:**
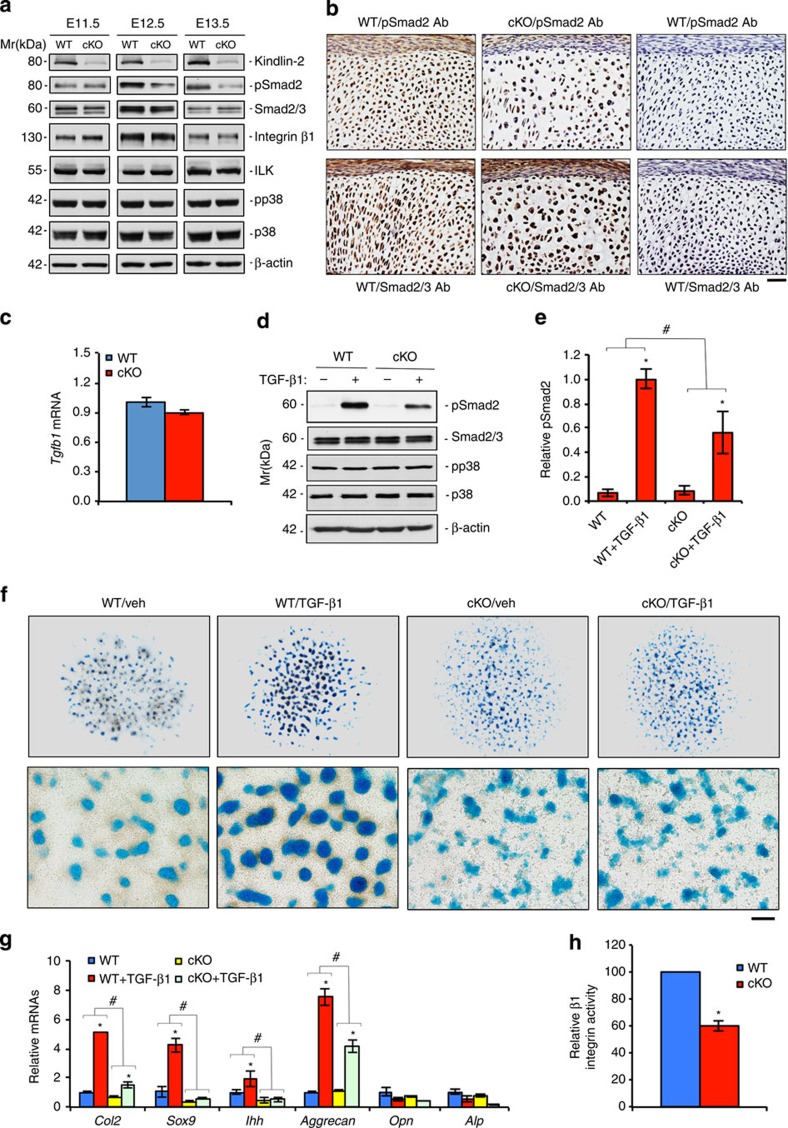
Kindlin-2 modulates TGF-β1 signalling during chondrocyte differentiation. (**a**) Protein extracts from E11.5–13.5 Cre-negative *Kindlin-2*^*fl/fl*^ (WT) and *Kindlin-2*^*Prx1*^ cKO (cKO) forelimbs were used for western blotting for the indicated proteins. (**b**) Immunohistochemistry. Sections of E12.5 WT and *Kindlin-2*^*Prx1*^ cKO forelimbs stained with antibodies against pSmad2 antibody (top) or total Smad2/3 (bottom). Scale bar, 40 μm. (**c**) Total RNA from E15.5 WT and *Kindlin-2*^*Prx1*^ cKO forelimbs were subjected to qPCR analysis for *Tgf*-β*1* mRNA, which was normalized to *Gapdh* mRNA. Results were expressed as mean±s.d. *N*=4. (**d**) Primary cells isolated from E12.5 WT and *Kindlin-2*^*Prx1*^ cKO forelimbs were treated with or without 2 ng ml^−1^ TGF-β1 for 30 min, followed by western blotting for the indicated proteins. (**e**) Quantitative data of pSmad2 from four independent experiments. *P<0.05 (versus no TGF-β1); #*P*<0.05 (TGF-β1-induced fold increases of pSmad2 levels in cKO versus those in WT), Student’s *t*-test. Results were expressed as mean±s.d. Experiments were repeated three times. (**f**,**g**) Primary mesenchymal progenitor cells isolated from E12.5 WT and *Kindlin-2*^*Prx1*^ cKO limbs were treated with 2 ng ml^−1^ TGF-β1 for 7 days, followed by alcian blue staining for chondrogenic nodules (F) or qPCR analysis (G). *P<0.05 (versus no TGF-β1); #*P*<0.05 (TGF-β1-induced fold increases of mRNA levels in cKO versus those in WT), Student’s *t*-test. Experiments were repeated three times in triplicate. Scale bar, 800 μm (top), 160 μm (bottom). Results were expressed as mean±s.d. (**h**) β1 integrin activation. Primary mesenchymal progenitors isolated from E12.5 WT and *Kindlin-2*^*Prx1*^ cKO limbs were subjected to measurement of β1 integrin activation as described in Methods. Experiments were repeated three times independently. *P<0.05 (versus WT), Student’s *t*-test. Results were expressed as mean±s.d.

TGF-β signalling is critical for skeletal development^11^,^12^,^13^,^14^,^17^,^18^. We examined whether the loss of Kindlin-2 in limb mesenchymal progenitor cells impairs TGF-β1 signalling. The results showed that Smad2 phosphorylation (pSmad2) was reduced in mutant limbs, compared to that of WT limbs, starting at E12.5 (Fig. 5a). The levels of total Smad2/3 proteins were not altered by the loss of Kindin-2 (Fig. 5a). The expression of β1 integrin and integrin-linked kinase (ILK), both Kindlin-2-interacting proteins^19^,^20^,^33^,^42^, were not altered in mutant limbs (Fig. 5a). Furthermore, the tyrosine phosphorylation of FAK, one of the key downstream effectors of integrin signalling, was not significantly altered in E13.5 *Kindlin-2*^*Prx1*^cKO versus WT limbs (Supplementary Fig. 5). Similarly, the levels of phospho-p38 and total p38 proteins, which belong to non-canonical TGF-β signalling^43^, were not affected by the loss of Kindlin-2 (Fig. 5a). The signal for pSmad2, but not total Smad2/3, was markedly reduced in both chondrocytes and perichondrial mesenchymal progenitors of mutant limbs (Fig. 5b). Notably, loss of Kindlin-2 did not reduce *Tgf*-β*1* mRNA expression in limbs (Fig. 5c). The levels of TGFβ R1 and TGFβ R2 proteins were similar in E13.5 *Kindlin-2*^*Prx1*^cKO versus WT limbs or cells (Supplementary Fig. 6). *In vitro* studies using primary mesenchymal progenitors from E12.5 limbs revealed that Kindlin-2 ablation (Supplementary Fig. 7) significantly impaired TGF-β1 induction of Smad2 phosphorylation (Fig. 5d,e). The results from limb micromass cultures showed that Kindlin-2 ablation severely impaired TGF-β1-induced chondrogenic nodule formation (Fig. 5f). Furthermore, the loss of Kindlin-2 significantly decreased the TGF-β1-induced expression of *Sox9*, *Col2*, *Ihh* (Indian hedgehog) and *Aggrecan* without affecting the levels of *Opn* (osteopontin) and *Alp* (alkaline phosphatase) mRNAs (Fig. 5g), two osteoblast markers. Finally, β1 integrin activation was significantly reduced in primary limb mesenchymal progenitor cells lacking Kindlin-2 compared with that in WT cells (Fig. 5h).

### *Kindlin-2* ^*Col2a1*^ cKO mice display dwarfism and kyphosis

**Figure 6:**
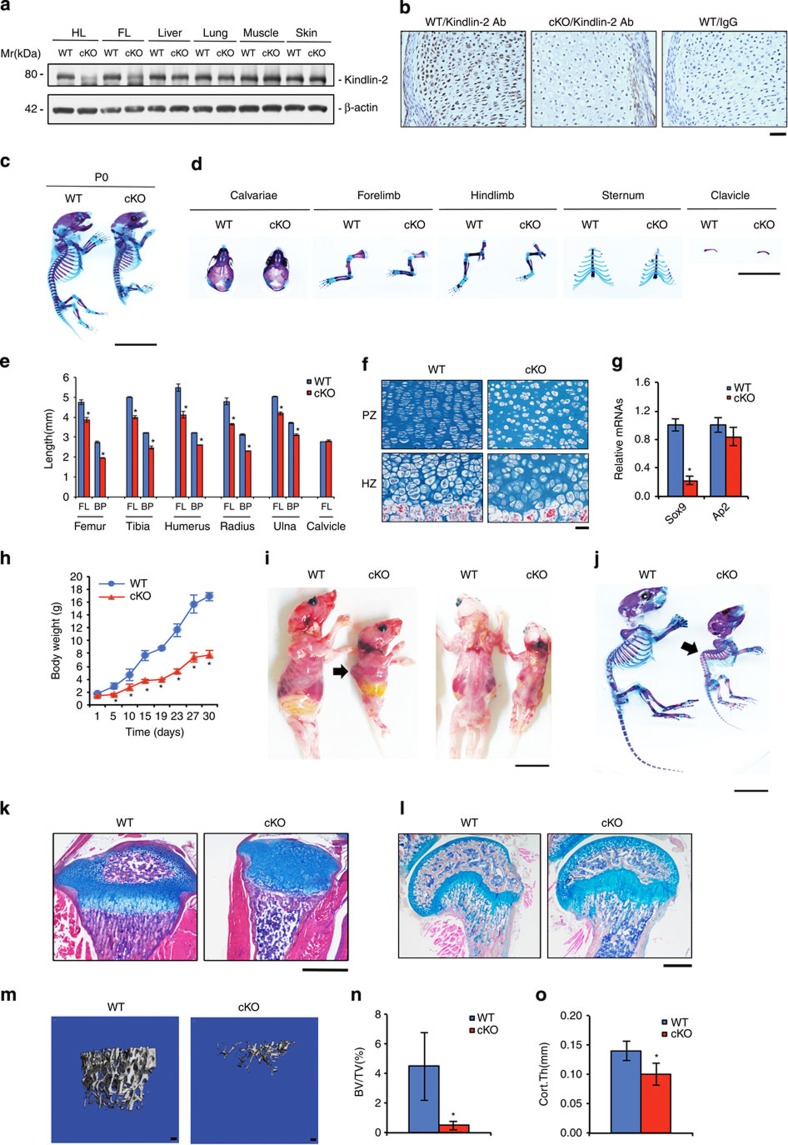
Deleting Kindlin-2 in chondrocytes causes multiple severe skeletal defects. (**a**) Protein extracts were isolated from the indicated tissues of P0 Cre-negative *Kindlin-2*^*fl/fl*^ (WT) and *Kindlin-2*^*Col2a1*^ cKO (cKO) mice, followed by western blot analysis for Kindlin-2 expression. β-Actin was used as a loading control. FL, forelimb; HL, hindlimb. (**b**) Immunohistochemistry. Sections of P0 WT and *Kindlin-2*^*Col2a1*^ cKO humeri stained with anti-Kindlin-2 antibody or normal IgG. Scale bar, 40 μm. (**c**) Alizarin red and alcian blue double stain of P0 WT and *Kindlin-2*^*Col2a1*^ cKO skeletons. Scale bar, 1 cm. (**d**) Calvaria, limb, sternum and clavicle from P0 WT and *Kindlin-2*^*Col2a1*^ cKO embryos. (**e**) Quantitation of P0 bone length. *N*=4, *P<0.05, versus WT, Student’s *t*-test. Scale bar, 1 cm. (**f**) Alcian blue stain of P0 humeral sections. Representative images of proliferative (PZ) and hypertrophic zone (HZ) chondrocytes show disrupted column formation in mutants. Original magnification, × 400. Scale bar, 40 μm. (**g**) Primary chondrocytes isolated from P3 ribcages of *Kindlin-2*^*Col2a1*^ cKO and WT were differentiated with 2 ng ml^−1^ TGF-β1 for 7 days, followed by qPCR analysis for *Sox9* and *Ap2* expression normalized to *Gapdh* mRNA. (**h**) Animal growth curves. *N*=4, *P<0.05, versus WT, Student’s *t*-test. Results were expressed as mean±s.d. **(i**) Gross appearance of P20 *Kindlin-2*^*Col2a1*^cKO and WT mice shows severe dwarfism and kyphosis. Scale bar, 1 cm. (**j**) Alizarin red and alcian blue double staining of P20 skeletons reveals reduced skeleton size and severe kyphosis in mutants. Scale bar, 1 cm. (**k**,**l**) Alcian blue stain of P17 (K) and P30 (L) tibial sections show delayed formation of the secondary ossification center and reduced subchondral bone in mutants. Scale bar, 400 μm. (**m**) Three-dimensional reconstruction from microcomputerized tomography (μCT) scans of femurs from 1-month-old female mice of the two genotypes. Scale bar, 100 μm. (**n**,**o**) Quantitative analyses of bone volume/tissue volume (BV/TV) and cortical bone thickness (Cort.Th) from L. *P<0.05 (versus WT), Student’s *t*-test, *N*=4. Results were expressed as mean±s.d.

To determine whether Kindlin-2 plays a direct role in chondrocytes, we conditionally, and selectively, deleted Kindlin-2 in chondrocytes by breeding *Col2a1-Cre* transgenic mice with *Kindlin-2*^*fl/fl*^ mice to obtain *Col2a1-Cre*; *Kindlin-2*^*fl/fl*^ mice (referred to as *Kindlin-2*^*Col2a1*^cKO hereafter). *Col2a1-Cre* transgenic mice expressing Cre recombinase under the control of the mouse *Col2a1* gene promoter are widely used to efficiently drive Cre expression in chondrocytes^44^. *Kindlin-2*^*Col2a1*^ cKO mice were born at the expected Mendelian frequency. Western blot analysis showed that Kindlin-2 was selectively reduced in mutant limbs (Fig. 6a). Immunohistochemical revealed that Kindlin-2 was essentially abolished in mutant chondrocytes (Fig. 6b). Limbs and vertebrae were shorter and the ribcage was smaller in mutant compared with WT mice (Fig. 6c–e). In contrast to *Kindlin-2*^*Prx1*^ cKO, mice lacking Kindlin-2 in chondrocytes did not exhibit any marked abnormalities in skull vault and clavicle (Fig. 6c–e), suggesting that intramembraneous ossification is unchanged in the mutant. Similar to *Kindlin-2*^*Prx1*^ cKO, ablating Kindlin-2 in chondrocytes disrupted column formation in long bones (Fig. 6f). It should be noted that the reduction of chondrocyte density in the long bone growth plate of *Kindlin-2*^*Col2a1*^ cKO was less severe than in *Kindlin-2*^*Prx1*^ cKO (compare Fig. 2b with Fig. 6f). Similar to *Kindlin-2*^*Prx1*^ cKO, deleting Kindlin-2 expression in chondrocytes downregulated *Sox9* mRNA without affecting the expression of the adipogenic gene, *Ap2* (adipocyte protein 2; Fig. 6g). However, after birth, all *Kindlin-2*^*Col2a1*^ cKO mice (>50) rapidly developed growth retardation and severe dwarfism (Fig. 6h,i). About 80% of the mice developed progressive kyphosis and died within 1 month of birth because of respiratory distress probably associated with compression of cervical and thoracic vertebrae from the kyphosis (Fig. 6i,j). Alcian blue staining of tibial sections showed delayed formation of the secondary ossification centre (Fig. 6k) and reduced subchondral bone (Fig. 6l) in mutants. Quantitative microcomputerized tomography (μCT) analysis of 1-month-old femur histomorphometric parameters showed that mutant mice have dramatic reductions in both trabecular and cortical bones (Fig. 6m-o). Collectively, these experiments establish that Kindlin-2 expression in chondrocytes plays an intrinsic role in the regulation of chondrocyte functions and chondrogenesis during prenatal and postnatal skeletal development.

## Discussion

In the study reported here, we demonstrated that Kindlin-2 functions in skeletal development by controlling endonchondral and intramembranous ossification processes. The severe skeletal abnormalities and biochemical defects in chondrocytes and their progenitors induced by the loss of Kindlin-2 suggest that Kindlin-2 serves as a major controlling factor in the regulation of the chondrocyte differentiation programme and chondrogenesis during prenatal and postnatal skeletal development.

This study represents the first systematic investigation into the functions of Kindlin-2 in chondrocyte differentiation and chondrogenesis, processes that are of broad clinical importance. The findings of our study can be summarized as follows. First, Kindlin-2 controls chondrocyte proliferation by regulating, at least in part, the expression of Ihh, a key factor for chondrocyte proliferation. Second, Kindlin-2 protects chondrocytes from apoptosis. Loss of Kindlin-2 greatly accelerates apoptosis of both proliferative and hypertrophic chondrocytes. Reduced cell proliferation and increased apoptosis contribute to reduced cellularity in the growth plates in the mutant mice. Third, loss of Kindlin-2 increases chondrocyte hypertrophy. Finally, Kindlin-2 is essential for chondrocyte column formation. Chondrocytes lacking Kindlin-2 are scattered throughout the growth plates rather than forming regular columns. Interestingly, deleting β1 integrin in chondrocytes in mice similarly disrupted column formation in growth plates^45^. Reduced chondrocyte numbers and disrupted chondrocyte organization together contribute to decreased longitudinal growth of long bones and digits, resulting in their shortening in mutants.

Importantly, we demonstrated that Kindlin-2 plays a critical role in the regulation of TGF-β signalling. The loss of Kindlin-2 reduced Smad2 phosphorylation, a key downstream molecular event of TGF-β signalling in limb chondrocytes *in vivo*. Of particular significance, TGF-β-induced Smad2 phosphorylation, chondrocyte differentiation gene expression, and chondrogenic nodule formation were all dramatically reduced in primary mesenchymal progenitor cells lacking Kindlin-2. These results clearly demonstrate that Kindlin-2 is crucial to the control of TGF-β signalling during the chondrogenic differentiation programme. Interestingly, while this study was in progress, Wei *et al*.^46^ reported that Kindlin-2 physically interacted with TGFβ R1 and activated Smad3 phosphorylation in human kidney tubular epithelial cells. Furthermore, a more recent study showed that Kindlin-1 controls TGF-β availability to regulate cutaneous stem cell proliferation^47^. Detailed molecular mechanisms through which Kindlin-2 modulates TGF-β signalling will be an important area of future studies.

Several important lines of evidence from this study, and studies by others, support the role of Kindlin-2 in regulating chondrocyte function and chondrogenesis by controlling, at least in part, the expression of Sox9, a master regulator of chondrocyte function. First, we found that the loss of Kindlin-2 dramatically reduced Sox9 expression at both the mRNA and protein levels in chondrocytes, as well as in limbs. Second, in our study, the overexpression of Kindlin-2 increased the levels of Sox9 mRNA and protein and activated *Sox9* gene promoter transcription. Third, we found that the forced expression of Sox9 through adenoviral vectors in primary mesenchymal progenitor cells largely restored the defects in chondrocyte differentiation gene expression and chondrogenic nodule formation induced by the loss of Kindlin-2. Finally, mice lacking Kindlin-2 in Prx1-expressing mesenchymal progenitors in the current study, and those lacking Sox9 in the same cell type^3^ share some striking cellular and skeletal abnormalities (for example, reduced chondrocyte proliferation and survival, increased hypertrophy, and severe limb shortening), suggesting that both factors may function in the same pathway. The fact that Kindlin-2 protein exists in the nuclei of chondrocytes and activates the *Sox9* gene promoter raises the possibility that Kindlin-2 may function as a transcription activator or co-activator for the *Sox9* gene, although it is also possible that Kindlin-2 upregulates the expression of some other protein(s) that in turn affect Sox9 expression. Future study will elucidate these possibilities.

Work from this study, and studies by others, suggest that the Kindlin-2-integrin signalling pathway plays an extremely important role in chondrogenesis. The selective deletion of Kindlin-2 in chondrocytes led to progressive dwarfism, severe kyphosis and low bone mass in our study, while, in another study, inactivation of β1 integrin in chondrocytes in mice resulted in a severe chondrodysplasia^45^. ILK, a Kindlin-2-binding protein, regulated chondrocyte shape and proliferation^48^ and mice lacking ILK in chondrocytes displayed a similar chondrodysplasia^49^. However, it is interesting to compare the phenotypes of the deletion of Kindlin-2 in Prx1-expressing mesenchymal progenitor cells (*Prx1-Cre; Kindlin-2*^*fl/fl*^ mice) in the current study with those of the deletion of β1 integrin (*Prx1-Cre; β1*^*fl/fl*^ mice)^50^ in the same cell type in mice. First, the skeletal defects exhibited by the *Prx1-Cre; Kindlin-2*^*fl/fl*^ mice were much more severe than those of the *Prx1-Cre; β1*^*fl/fl*^ mice. *Prx1-Cre; Kindlin-2*^*fl/fl*^ mice died immediately after birth while *Prx1-Cre; β1*^*fl/fl*^ mice survived at birth and beyond. Second, *Prx1-Cre; Kindlin-2*^*fl/fl*^ mice displayed severe defects in both intramembranous and endochondral ossification, while no defects in intramembranous ossification were reported in *Prx1-Cre; β1*^*fl/fl*^ mice, although they did exhibit less severe defects in endochondral ossification, including shortened long bones. These findings suggest that Kindlin-2 controls intramembranous ossification independent of β1 integrin activation and/or a functional compensation of β3 integrin for the loss of β1 integrin in the head mesenchyme. Thus, while the current study supports an important role of Kindlin-2 in regulation of the integrin signalling pathway in skeletal development, it suggests that Kindlin-2 also plays pivotal roles in the regulation of other major developmental pathways (for example, Sox9 expression and TGF-β signalling) in the skeleton. The genetic models and findings described in this paper will be instrumental for future studies aimed at dissecting the integrin-dependent and independent functions of Kindlin-2 during different stages of skeletal development and homeostasis.

## Methods

### Generation of *Kindlin-2* ^*fl/fl*^ Mice

To generate *Kindlin-2*^*fl/fl*^ mice, a *Kindlin-2* targeting vector was generated as depicted in Supplementary Fig. 1a. This targeting vector was linearized and introduced into R1 embryonic stem (ES) cells via electroporation. G418-resistant ES cell clones were screened for homologous recombination by Southern blotting (Supplementary Fig.1b). Of 180 ES cell clones analysed, 10 had homologous recombination. Two of the ES cell clones were injected into blastocysts from C57BL/6 mice, resulting in generation of chimeric mice, five of which were almost 100% agouti males. We obtained germ-line transmission of the floxed allele (*Kindlin-2*^*fl/+*+neo^) by crossing the chimeric mice with C57BL/6 breeders. To avoid potential interference from the pGKneo cassette, we crossed the mice with flippase deleter mice^51^ to generate floxed (without the neo cassette) heterozygous mice (*Kindlin-2*^*fl/+*^). The *Kindlin-2*^*fl/+*^mice were intercrossed to generate mice that are homozygous floxed (*Kindlin-2*^*fl/fl*^). Both heterozygous and homozygous animals were viable and fertile and showed no noticeable phenotypic changes. All mice used in this study have been crossed with normal C57BL/6 mice for at least eight generations to minimize potential effects of genetic background on skeletogenesis and homeostasis. All research protocols were approved by the respective Institutional Animal Care and Use Committees (IACUC) of University of Pittsburgh, University of California, San Diego or Rush University.

### Bone histology and immunohistochemistry

Embryos and neonates were stained with alcian blue and alizarin red as previously described^52^. In brief, embryos and neonates were eviscerated and fixed in 100% ethanol for 4 days and transferred to acetone. After 3 days, they were rinsed with water and stained for 10 days in staining solution containing 1 vol 0.1% alizarin red S (Sigma, St. Louis, MO) in 95% ethanol, 1 vol 0.3% alcian blue 8GX (Sigma) in 70% ethanol, 1 vol 100% acetic acid and 17 vol ethanol. After rinsing with 95% ethanol, specimens were kept in 20% glycerol/1% KOH at 37 °C for 24 h and then at room temperature until the skeletons became clearly visible. For storage, specimens were transferred into 50%, 80%, and finally, 100% glycerol. For histology, bone tissues were fixed in 10% formalin at 4 °C for 24 h, decalcified in 10% EDTA (pH 7.4) for 10–14 days and embedded in paraffin. Bone sections were used for haematoxylin and alcian blue staining using our standard protocols. Fixed non-demineralized femurs were used for microcomputerized tomography (μCT) analysis in the Department of Biochemistry of Rush University Medical Center using a μCT35 (SCANCO Medical AG), as previously described^53^. Following killing, femurs were isolated and fixed in 70% ethanol for 2 days. Non-demineralized femurs from each group were scanned and measured by μCT (μCT35, SCANCO Medical AG, Wayne, PA) with an isotropic resolution of 7.0 μm, following the standards of techniques and terminology recommended by the American Society for Bone and Mineral Research^54^. For trabecular bone parameters, transverse slices were obtained in the region of interest in the axial direction from the trabecular bone 0.1 mm below the growth plate (bottom of the primary spongiosa). Contours were defined and drawn close to the cortical bone. The trabecular bone was then removed and analysed separately. A three-dimensional analysis was performed on 250 trabecular bone slices. A 1.75-mm section was used to obtain mid-femoral cortical bone thickness. The analysis of the specimens involved the following bone measurements—bone volume fraction/total tissue volume (%) and cortical thickness (Cort.Th). For immunohistochemistry, 5-μm sections were stained with antibodies against Kindlin-2 (Abcam, cat#: ab74030, 1:400), active caspase-3 (Abcam, cat# ab2302, 1:300), pSmad2 (Cell Signaling Technology, cat#: 8828S, 1:300), total Smad2/3 (Cell Signaling Technology, cat#: 8685S, 1:500) or control IgG using the EnVision^+^System-HRP (DAB) kit (Dako North America Inc.) according to the manufacturer’s instruction.

### Immunofluorescence and confocal analysis

Cells were seeded and cultured on sterile glass cover slips in six-well plates. Twenty-four hours later, cells were fixed in 10% formaldehyde for 30 min at 37 °C. Cells were permeabilized with 0.2% Triton-X-100 containing 4′,6-diamidino-2-phenylindole for 5 min, and blocked with 2% bovine serum albumin for 1 h. The cells were then stained with a primary antibody (mouse anti-Kindlin-2, diluted 1:300) overnight at 4 °C. After washing, the cells were incubated with anti-mouse Alexa Fluor 594 (Invitrogen) secondary antibodies (1:300) for 1 h at room temperature. Cells were then imaged using a confocal microscope (SP2-AOBS Leica Microsystems).

### *In vivo* cell proliferation and TUNEL staining

BrdU staining was used to measure cell proliferation as previously described^55^. In brief, pregnant mice (E14.5) were injected intraperitoneally with 100 μg bromodeoxyuridine (BrdU)/12 μg fluorodeoxyuridine per gram of body weight 3 h before killing. After killing, embryos were removed and genotyped. Humeri of WT and *Kindlin-2*^*Prx1*^ cKO mice were isolated and sectioned at 10 μm. To identify actively proliferating chondrocytes, nuclei that have incorporated BrdU were detected using a Zymed BrdU immunostaining kit according to the manufacturer’s instruction. BrdU-positive cells were counted and normalized to the total cell numbers in the same area. Cell survival was evaluated using the ApopTag Peroxidase *In Situ* Apoptosis Detection Kit (Millipore) as previously described^56^,^57^. This method is based on the classical TUNEL assay to examine apoptosis by detecting DNA fragmentation. Sections of WT and *Kindlin-2*^*Prx1*^ cKO humeri were prepared and stained using the kit according to the manufacturer’s instruction. Cells were counterstained with haematoxylin. All positive (brown) and negative (blue) nuclei were counted. Apoptotic cells were normalized to the total cells from the same area.

### qPCR and western blot and *in situ* hybridization

RNA isolation and reverse transcription (RT) have been previously described^58^. In brief, total RNAs were isolated using the Trizol reagent (Invitrogen) following the manufacturer’s protocol. RT was performed using 1 μg of denatured RNA and 100 pmol of random hexamers (Applied Biosystem, Foster, CA) in a total volume of 25 μl containing 12.5 U MultiScribe reverse transcriptase (Applied Biosystem). Quantitative real-time RT-PCR (qPCR) analysis was performed to measure the relative mRNA levels using the SYBR Green kit (Bio-Rad Laboratories Inc.). Samples were normalized to *Gapdh* expression. The DNA sequences of primers used for qPCR are summarized in Supplementary Table 1. Western blot analysis was performed as previously described^59^. In brief, protein extracts were fractionated on a 10% SDS–polyacrylamide gel electrophoresis gel and transferred onto nitrocellulose membranes (Schleicher & Schuell, Keene, NH). The membrane was blocked in 5% non-fat milk in Tris-buffered saline/Tween 20 buffer; probed with primary antibodies, followed by incubation with secondary antibodies conjugated with horseradish peroxidase; and visualized using a Western Blotting Detection Kit (GE Healthcare, cat#: RPN2106). Antibodies used for western blotting in this study were Kindlin-1 (Sigma, SAB4200465, 1:1,000), Kindlin-3 (Cell Signaling, Cat# 13,843, 1:300), TGFβ R1 (Santa Cruz, sc-9048, 1:500), TGFβ R2 (Santa Cruz, sc-400, 1:500), pFAK (Santa Cruz, sc-11765, 1:300), Sox9 (Santa Cruz Biotechnology, sc-20095, 1:500), pSmad2/3 (Cell Signaling Technology, cat#: 8828S, 1:500), total Smad2/3 (Cell Signaling Technology, cat#: 8685S, 1:500), integrin β1 (BD, cat# 610468, 1:3,000), ILK (Cell Signaling, cat# I0783, 1:1,000), p38 MAPK (Cell Signaling, cat# 9,212, 1:1,000), phospho-p38 MAPK (Cell Signaling, cat# 9,216, 1:1,000), (Sigma-Aldrich, cat#: T-7451, 1:1,000), tubulin (Sigma-Aldrich, cat#: T-7451, 1:5,000), β-actin (Sigma-Aldrich, cat#: A5316, 1:4,000). The Kindlin-2 antibody (1:1,000) has been previously described^19^. Secondary anti-rabbit (1:5,000) or anti-mouse (1:5,000) antibodies conjugated with horseradish peroxidase were from Santa Cruz Biotechnology Inc. Images of western blots are provided as Supplementary Figs 8 and 9. Five-μm bone sections were mounted onto polylysine-treated slides and subjected to *in situ* hybridization using a protocol provided by the Center for Musculoskeletal Research, University of Rochester (http://www.urmc.rochester.edu/musculoskeletal-research/core-services/histology/protocols.cfm). The vectors for preparing the *Sox9* and *Ihh* probes were kindly provided by Dr Matthew J. Hilton (University of Rochester Medical Center). Antisense riboprobe syntheses were prepared using the DIG RNA Labeling Mix from Roche (Cat#: 11277073910).

### DNA constructs and transfection and infection

pCMV/β-gal and pCMV/Kindlin-2 were previously described^19^. p2.8Sox9-luc was kindly provided by Professor Elazar Zelzer of the Weizmann Institute of Science. For transfection, the cells were plated on 35-mm dishes at a density of 5 × 10^4^ cells cm^−2^. After 24 h, the cells were transfected with LipofectAMINE 2,000 (Invitrogen) according to the manufacturer’s instructions. Each transfection contained 0.25 μg of the indicated reporter plasmids plus 1 ng of pRL-SV40, containing a complementary DNA for Renilla Reformis luciferase to control for transfection efficiency. The cells were harvested and assayed using the Dual Luciferase Assay Kit (Promega, Madison, WI) on a Module Microplate Multimode Reader (Turner Biosystem, Sunnyvale, CA). For all transfection experiments, the amount of plasmid DNA was balanced as necessary with pCMV/β-gal such that the total DNA was constant for each group. Adenoviral vectors for enhanced green fluorescent protein (Ad/EGFP) and Kindlin-2 (Ad/Kindlin-2) were previously described^20^. Ad/Cre was kindly provided by Dr Yifan Dai of the Nanjing Medical University. Ad/Sox9 was purchased from Vector BioLabs (Philadelphia, PA). Cells were infected with adenovirus as previously described^58^. In briefly, virus was added to cells in 1% fetal bovine serum (FBS) and incubated for 1 h at 37 °C. Dishes were rotated every 5 min for the first 15 min to ensure that all of the cells were exposed to virus. After 1 h, media were aspirated, and cultures were rinsed twice with serum-free medium, and then fresh media supplemented with 10% FBS were added to the dishes. The amount of adenovirus was balanced as necessary with Ad/EGFP such that the total amount was constant in each group.

### Isolation of limb MSCs and micromass culture

Isolation of limb mesenchymal cells was performed as previously described^11^. In brief, limb buds from E12.5 or E13.5 embryos were isolated, dissociated into single cell suspension by digesting with 1 mg ml^−1^ collagenase D (Roche Applied Sci., Cat# 11088858001) for 3 h in a 37-°C incubator and filtered to remove undigested tissues. The cells were pelleted by centrifugation at 1,000 r.p.m. for 5 min, resuspended and reconstituted at a density of 1 × 10^7^ cells ml^−1^ in F12:DMEM (1:1) media supplemented with 10% FBS and antibiotics. For micromass cultures, 1 × 10^5^ cells in 10 μl were dropped into a dish and allowed to attach for 1 h in a 37-°C incubator. The cells were then flooded with the F12:DMEM (1:1) media supplemented with 10% FBS and antibiotics, 50 μg ml^−1^ ascorbic acid and 10 mM β-glycerol phosphate for the indicated times, followed by alcian blue staining, western blot or qPCR analyses.

### Measurement of β1 integrin activation

Integrin activation was analysed based on a protocol that we have previously described^60^. In brief, active β1 integrin was detected by incubating the mesenchymal cells isolated from WT and *Kindlin-2*^*Prx1*^ cKO limbs with a rat anti-β1 integrin antibody (9EG7) that specifically recognizes the active form of β1 integrin (BD Pharmingen). The cells were washed three times with PBS containing 5 mg ml^−1^ bovine serum albumin and then incubated with FITC-conjugated anti-rat IgG at 4 °C for 30 min. Total β1 integrin was detected by incubating the cells with Alexa Fluor 647-conjugated anti-β1 integrin antibody that recognizes total β1 integrin (BioLegend catalogue number 102,214) at 4 °C for 30 min. The levels of the active and total β1 integrin were quantified using a BD FACS Calibur flow cytometer. The integrin activation index is defined as the mean fluorescence intensities of active β1 integrin divided by the mean fluorescence intensities of β1 integrin. The effect of loss of Kindlin-2 on β1 integrin activation was assessed by comparing the activation index in mutant cells with that of Cre-negative (WT) cells (normalized to 100%).

### Statistical analysis

The data were analysed using the GraphPad Prism software (4.0). Student’s’ *t*-test was used to test for differences between two groups of data. Results were expressed as mean±s.d. Differences with *P*<0.05 were considered statistically significant. All experiments were repeated at least three times.

## Additional information

**How to cite this article:** Wu, C. *et al*. Kindlin-2 controls TGF-β signalling and Sox9 expression to regulate chondrogenesis. *Nat. Commun.* 6:7531 doi: 10.1038/ncomms8531 (2015).

## Supplementary Material

Supplementary Information Supplementary Figures 1-9 and Supplementary Table 1
