# Supplementary material for: Kindlin-2 controls TGF-β signalling and Sox9 expression to regulate chondrogenesis
Source: Nat Commun. 2015 Jul 7;6:7531. doi: 10.1038/ncomms8531 (PMC4498276; doi:10.1038/ncomms8531)
Supplement: Supplementary Information — Supplementary Figures 1-9 and Supplementary Table 1 [file ncomms8531-s1.pdf]

SUPPLEMENTARY INFORMATION

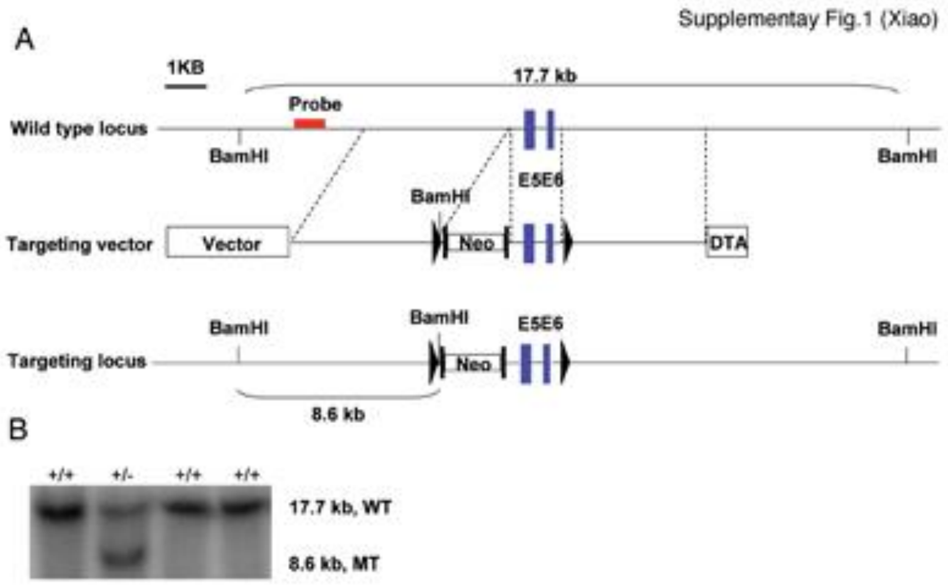

Supplementary Figure 1. Generation of a conditional allele of the *Kindlin-2* gene. (**A**) A restriction map of the relevant genomic region of Kindlin-2 (top), the targeting construct (middle), and the mutated locus after recombination (bottom) are shown. The blue rectangles indicate the targeted exons 5 and 6; black triangles indicate LoxP sites, and blue rectangles indicate frt sites; DTA, Diphtheria Toxic A chain gene; Neo, Neomycin resistance gene. (**B**) Detection of WT and mutated alleles (MT). DNA from electroporated embryonic stem (ES) cells was digested with BamHI and analyzed by Southern blot with the probe shown in A. The 17.7 kb and 8.6 kb bands represent WT and mutated alleles (MT), respectively.

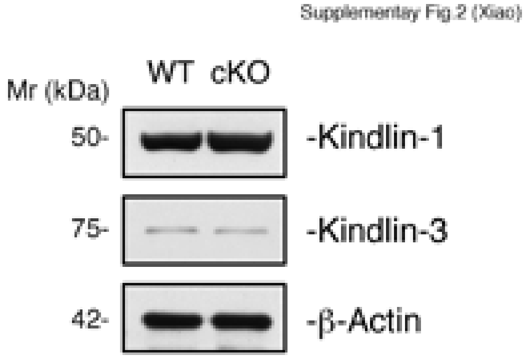

Supplementary Figure 2. Loss of Kindlin-2 does not alter the levels of KIndlin-1 and KIndlin-3 proteins. Protein extracts were isolated from E13.5 Cre-negative *Kindlin-2*$^{fl/fl}$ (WT) and *Kindlin-2*^{Prx1} cKO limbs, followed by western blot analysis for Kindlin-1 and Kindlin-3 expression. β-Actin was used as a loading control. Experiments were repeated 3 times.

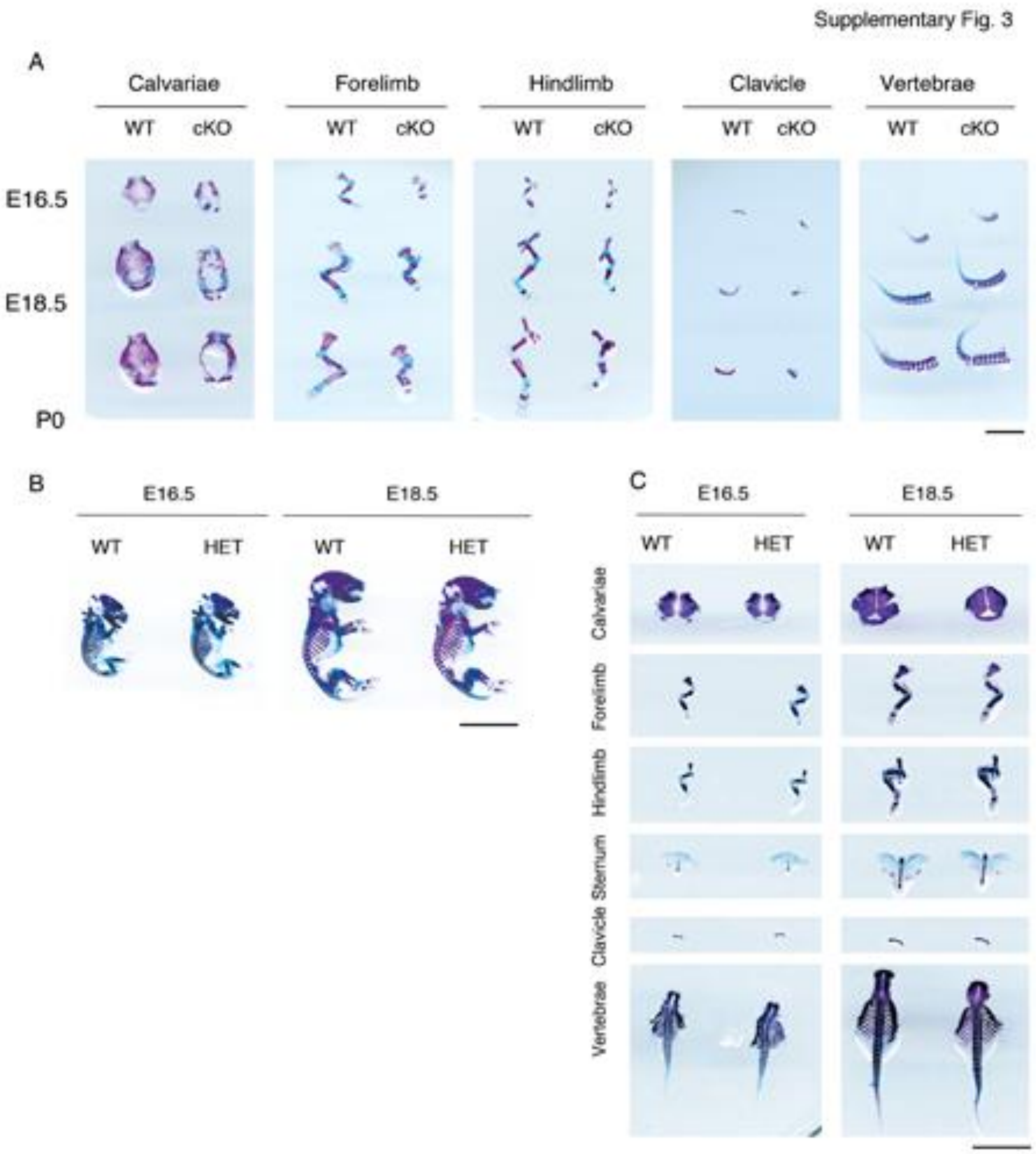

Supplementary Figure 3. Staining of *Kindlin-2^{Prx1}* cKO, *Prx1-Cre; Kindlin-2$^{fl/+}$*, and WT skeletons. (**A**) Alizarin red and alcian blue double stain of *Kindlin-2^{Prx1}* cKO (cKO) and Cre-negative *Kindlin-2$^{fl/fl}$* (WT) mice showing lack of skull vault, shortened and broadened limbs and sternum, hypoplastic clavicle, and impaired elongation of distal

digits in *Kindlin-2*^{Prx1} cKO mice. N=5. Scale bar: 0.5 cm. (**B**) Alizarin red and alcian blue double stain of E16.5 and E18.5 *Prx1-Cre; Kindlin-2*$^{fl/+}$ (HET) and WT skeletons, showing no marked differences between the two genotypes. N=5. Scale bar: 1 cm. (**C**) Calvaria, forelimb, hindlimb, sternum, and clavicle from E16.5 and E18.5 *Prx1-Cre; Kindlin-2*$^{fl/+}$ (HET) and WT embryos. N=5. Scale bar: 1 cm.

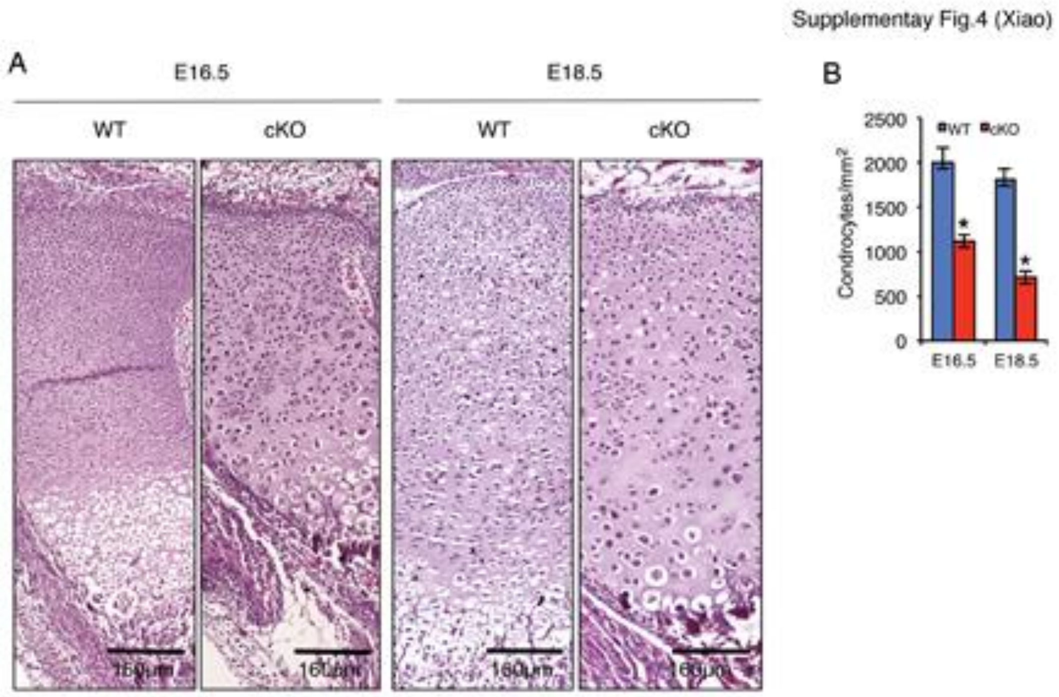

Supplementary Figure 4. Reduced cellularity, abnormal cell morphology, and disrupted column formation in *Kindlin-2*^{Prx1} cKO tibial growth plates.
(**A**) Hematoxylin staining of E16.5 and E18.5 tibiae from of *Kindlin-2*^{Prx1} cKO (cKO) and Cre-negative *Kindlin-2*$^{fl/fl}$ (WT) mice. Representative images from each group are shown. Original magnification: 100x. Scale bar: 160 μm. (**B**) Quantitation of chondrocyte densities of E16.5 and E18.5 tibial growth plates. N=4, *p<0.05, versus WT, Student's *t*-test. Results were expressed as mean ± standard deviation.

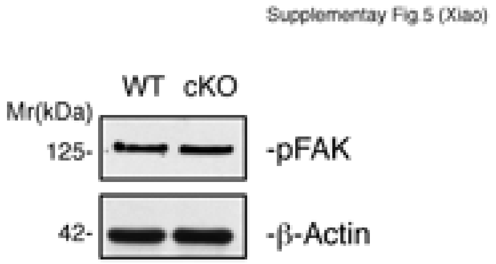

Supplementary Figure 5. Loss of Kindlin-2 does not reduce the level of pFAK protein in limbs. Protein extracts were isolated from E13.5 Cre-negative *Kindlin-2*$^{fl/fl}$ (WT) and *Kindlin-2*^{Prx1} cKO limbs, followed by western blot analysis for Kindlin-1 and Kindlin-3 expression. β-Actin was used as a loading control. Experiments were repeated 3 times.

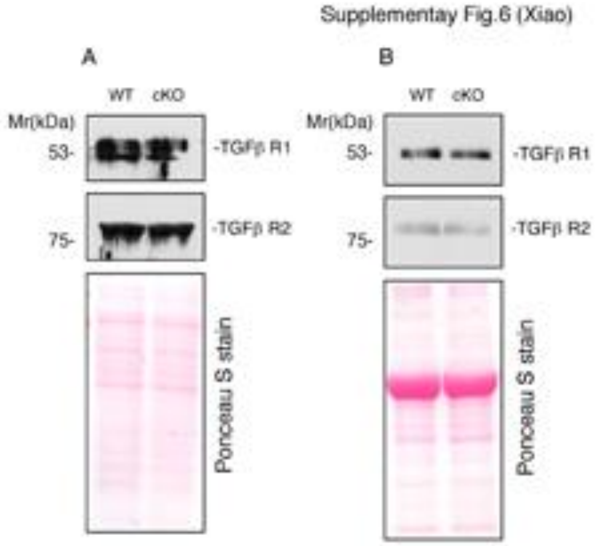

Supplementary Figure 6. Loss of Kindlin-2 does not reduce the levels of TGFβ R1 and TGFβ R2 proteins. Protein extracts were isolated from limbs (A) or primary limb mesenchymal progenitors (B) of E13.5 Cre-negative *Kindlin-2*$^{fl/fl}$ (WT) and *Kindlin-2*^{Prx1} cKO mice, followed by western blot analysis for TGFβ R1 (top) and TGFβ R2 (middle) expression. Ponceau S stain of the blots immediately after transferring (bottom). Experiments were repeated 3 times.

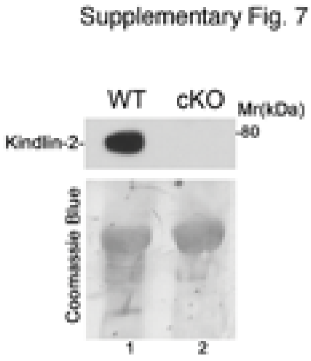

Supplementary Figure 7. Kindlin-2 protein is essentially abolished in primary mesenchymal progenitors from E12.5 *Kindlin-2*^{Prx1} cKO limbs. Protein extracts were isolated from primary mesenchymal progenitors of E12.5 Cre-negative *Kindlin-2*$^{fl/fl}$ (WT) and *Kindlin-2*^{Prx1} cKO limbs, followed by western blot analysis for Kindlin-2 expression. Experiments were repeated 3 times.

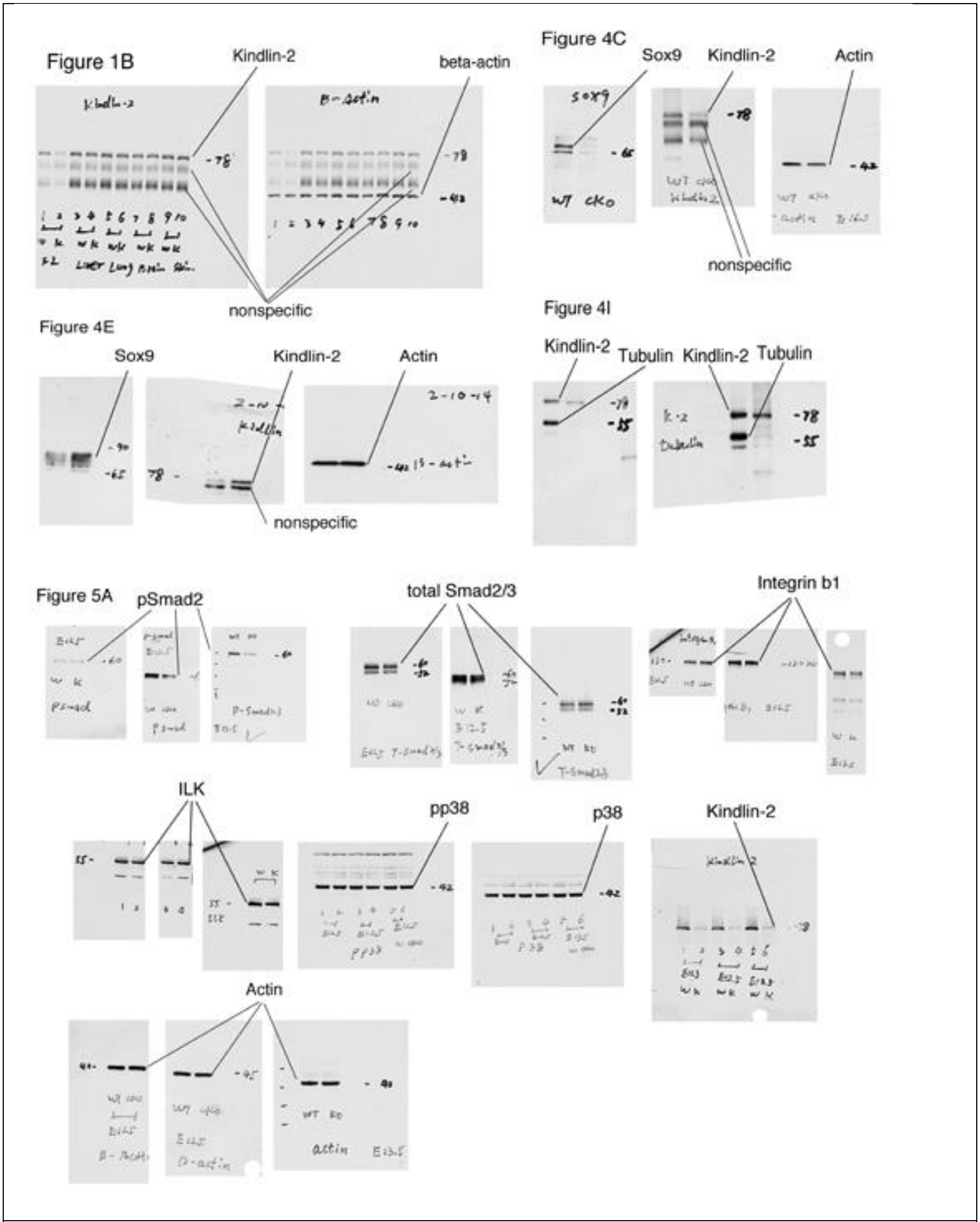

Supplementary Figure 8. Western blots (1)

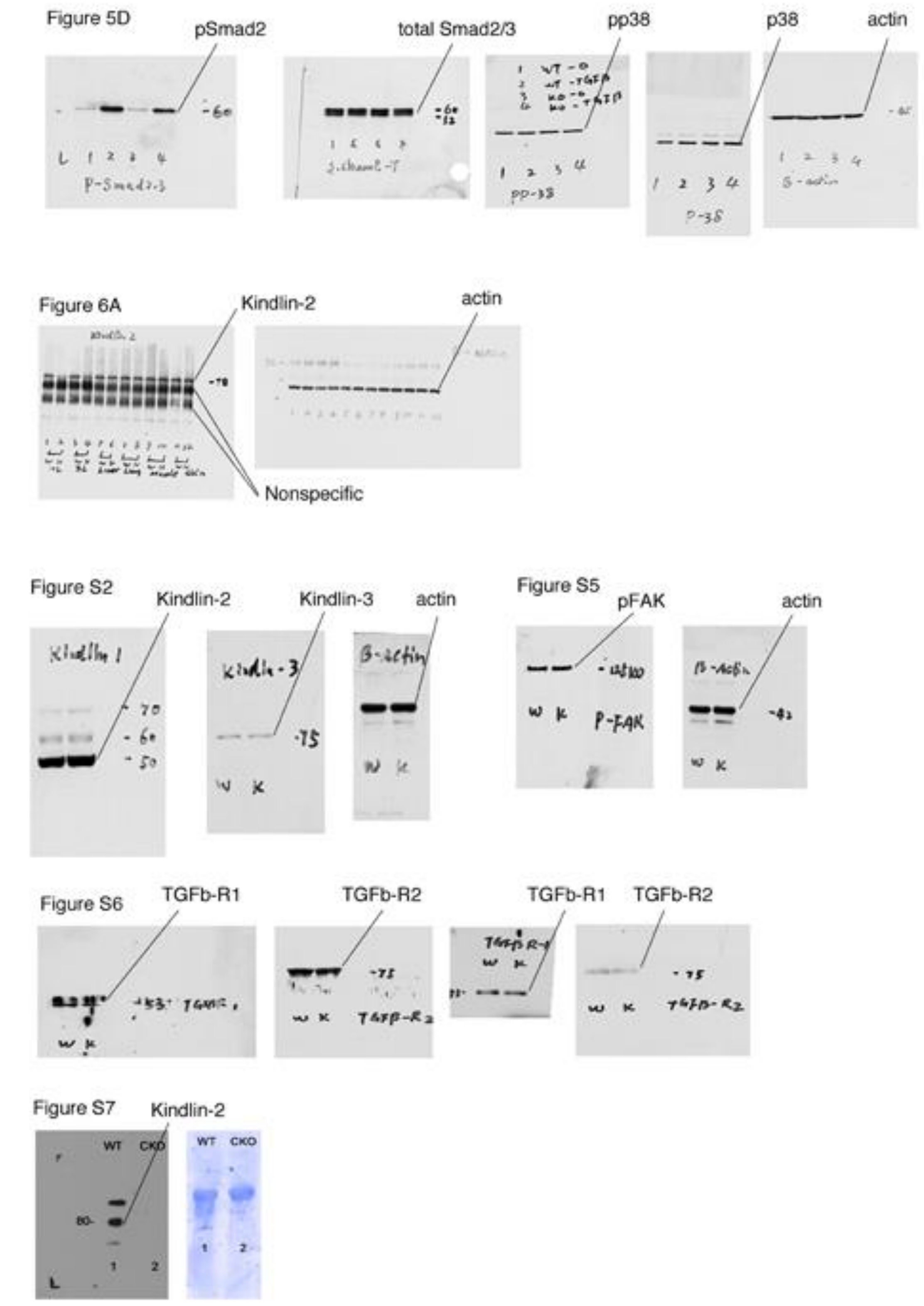

Supplementary Figure 9. Western blots (2)

Supplementary Table 1 : Real-time Polymerase Chain Reaction (PCR) Primers

Name	5’ primer	3’ primer
Actin	TCCTCCTGAGCGCAAGTACT CT	CGGACTCATCGTACTCCTGCT T
Aggrecan	AGGACCTGGTAGTGCGAGTG	GCGTGTGGCGAAGAA
Cadherin	AGCGCAGTCTTACCGAAGG	TCGCTGCTTTCATACTGAACTTT
Ap2	GATGAAATCACCGCAGACGA CAGGA	CACCACCAGCTTGTCACCATCTC G
Col2a1	CCACACCAAATTCCTGTTCA	ACTGGTAAGTGGGGCAAGAC
Gapdh	CAGTGCCAGCCTCGTCCCGT AGA	CTGCAAATGGCAGCCCTGGTGAC
Ihh	TGGACTCATTGCCTCCCAGA	CAAAGGCTCAGGAGGCTGGA
Kindlin-2	TGGACGGGATAAGGATGCCA	TGACATCGAGTTTTTCCACCAAC
Runx2	TAAAGTGACAGTGGACGGTC CC	TGCGCCCTAAATCACTGAGG
Sox9	CCACGGAACAGACTCACATC TCTC	CTGCTCAGTTCACCGATGTCCAC G
Tgf-β1	GGATACCAACTATTGCTTCAG CTCC	AGGCTCCAAATATAGGGGCAGGG TC

Ap2=adipocyte protein 2; Ihh=Indian hedgehog; TGF=transforming growth factor
